# Therapeutic management of buying/shopping disorder: A systematic literature review and evidence-based recommendations

**DOI:** 10.3389/fpsyt.2022.1047280

**Published:** 2022-11-03

**Authors:** Octavian Vasiliu

**Affiliations:** Department of Psychiatry, Dr. Carol Davila University Emergency Central Military Hospital, Bucharest, Romania

**Keywords:** compulsive buying, shopping addiction, behavioral addictions, psychotherapy, antidepressants, mood stabilizers, atypical antipsychotics

## Abstract

The prevalence of buying/shopping disorder (B/SD) has been increasing in the last two decades, and this disorder has a substantial negative impact on general functioning and quality of life. Therefore, a systematic review of the studies dedicated to the efficacy and tolerability of therapeutic interventions, both psychological and pharmacological, might help clinicians to decide on the most evidence-based treatment for these patients. In order to further increase the clinical usefulness of the current review, GRADE-based recommendations were formulated, where enough evidence was found to support such an approach. A number of five electronic databases were searched for single case reports, case series, open-label and double-blind, placebo/active intervention-controlled trials, but other secondary reports (i.e., systematic reviews and meta-analyses) were also included in this analysis. Studies with unspecified designs or those that do not report either qualitatively or quantitively the evolution of B/SD core manifestations were excluded. All data included in the secondary analysis were evaluated using the Joanna Briggs Institute critical appraisal checklists. A total number of 24 manuscripts (i.e., 12 clinical trials, eight case reports, and four reviews) were included. Most of the reviewed studies were of moderate quality, representing a certain limitation of this review and preventing the formulation of high-validity recommendations. Psychotherapy, especially cognitive behavioral therapy (CBT) seems to be the main intervention supported by the current evidence, followed by the combination of antidepressants and CBT, and serotoninergic antidepressants as monotherapy. There is an obvious need to further develop good-quality trials with a more significant number of participants with B/SD and longer follow-up periods.

## Introduction

“Compulsive shopping/buying” (CS/CB), also known as “buying/shopping dependence/addiction,” “pathological buying,” or “oniomania” represents a behavioral addiction defined by excessive financial investments (either online or in the real world), which cause distress or significant dysfunctions to the patients (1, 2).

**Figure 1:**
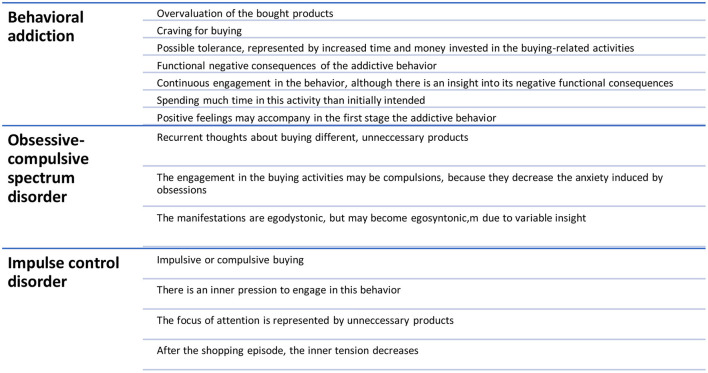
Compulsive shopping disorder—clinical hypotheses.

While some authors consider CS/CB an addiction, others include this condition in the “impulse-control disorders” category or within the obsessive-compulsive disorders (OCD) spectrum (Figure 1) (1–3). For the objectives of this review, the term “buying/shopping disorder” (B/SD) will be used in order to avoid insufficiently proven categorizations and to highlight that reviewed therapies are dedicated to patients with significant impairments due to their behaviors, not to the shoppers who may occasionally overbuy and later regret their investments. This terminology is far from being redundant because of the continuum model several authors suggested, a model that states the difference between normality and pathology is based on the frequency and intensity of the problematic behavior and on its various dysfunctional consequences (e.g., professional, social, academic, legal, financial) (4). Also, the DSM-5 uses the same terminology, i.e., “gambling disorder” and “Internet gaming disorder” for other pathologies with intricate, addictive, compulsive, and impulsive mechanisms (5).

B/SD may be conceptualized as a behavioral addiction (BA) due to the shared core manifestations between this pathology and substance use disorders (SUDs), and also based on the presumed common hyperstimulation of the dopamine reward system (6). The overvaluation of the shopping process/objects purchased was considered similar to the high importance attributed to the drugs by patients with SUD. Also, impulsivity and lack of inhibition during drug use or shopping are shared features of these two disorders (6). This rationale is similar to other BAs, e.g., food addiction, physical exercise addiction, Internet addiction, etc. (7). Cultural factors might contribute to the pathogenesis of B/SD because it has been reported mainly in developed countries (3), but this could be a bias due to the higher income and increased accessibility to the products when compared to low and medium-income countries. According to the tenth version of the International Classification of Mental Disorders (ICD-10), B/SD may be considered an “impulse control disorder, not otherwise specified” because of the repeated acts of buying without a well-defined motivation, lack of control over these behaviors, and the presence of functional negative consequences (8). There is no mention of the B/SD in the last edition of the same classification (9).

Diagnostic criteria for B/SD have been created (10), with the accent being placed upon irresistible impulses and frequent preoccupation with buying, but also on discomfort, and time-consuming or negative financial and social consequences, in the absence of mania or hypomania. Also, a type of “co-dependent buyer” has been described in the literature, and this term is reserved for those who are driven by the desire to purchase items for other individuals based on their wish to obtain approval, to be validated emotionally, or to avoid rejection (11).

B/SD has been diagnosed as a comorbidity in patients who have SUDs, mood disorders, personality disorders, or obsessive-compulsive spectrum disorders (OCSD) (1). Compulsive hoarding, anxiety disorders, and various other impulse control disorders may also be diagnosed in patients with B/SD (12). This pathology has been associated with a high prevalence of suicidal ideation, reaching 18.4% in a study that included 4,404 patients with different behavioral addictions (out of which 158 presented B/SD) (13). Suicide attempts were detected in 7.6% of the B/SD patients, and female gender, lack of family support, and unemployment were associated with the greater risk for suicide (13). According to a case series (*N* = 20 patients) that used structured evaluation for patients with problematic buying behavior, 95% had lifetime diagnoses of major mood disorders, 80% associated anxiety disorders, 40% presented impulse control disorders, and 35% also suffered from eating disorders (14). Also, it was observed that first-degree relatives presented a high prevalence of mood disorders (14).

The prevalence of B/SD in the United States was estimated to be 5%, and the most vulnerable individuals to B/SD onset were adolescents and young adults (1). Women are dominant in this group, with a proportion reaching 80% in clinical samples (10). Some authors consider this difference artificial, based on research that women are more open to admitting their pathological behavior (15). A meta-analysis with 49 articles included focused on the prevalence of CS in 16 countries (*N* = 32,000) and reported a 4.9% pooled prevalence in adults, with higher rates in university students (up to 11.5%) (16). The prevalence of CS in shopping-specific populations (e.g., individuals shopping in supermarkets or malls) had a value of up to 27.8% (16). European surveys reported an increase in B/SD in the adult population over the last two decades, indicating the necessity to develop adequate strategies for early detection and treatment (17).

Several instruments have been created for the structured evaluation of the B/SD, e.g., the Compulsive Buying Scale (CBS), Yale-Brown Obsessive-Compulsive Scale-Shopping version (YBOCS-SV), Pathological Buying Screener (PBS), and Compulsive Buying Follow-up Scale (CBFS) (18–22). CBS includes seven items representing activities, motivations, and feelings associated with buying, and a score ≤ -1.34 indicates a possible compulsive nature of this behavior (18). Another version of the CBS included 29 items, distributed on five factors (“tendency to spend,” “compulsion/drive to spend,” “feelings about shopping and spending,” “dysfunctional spending,” and “post-purchase guilt”), and allows the classification of individuals across a compulsive buying spectrum- from normal, recreational, or borderline, to compulsive, or addictive shopping patients (19). YBOCS-SV was designed to evaluate the cognitions and behaviors related to compulsive buying, instead of assessing obsessions and compulsion, as in the original YBOCS, and it includes 10 items, with a total score interval between 0 and 40 (20). PBS has 20 items, but another, 13-item version with two factors (“loss of control/consequences” and “excessive buying behavior”), has also been created (21). CBFS is a self-administered instrument with six self-reported items that evaluate aspects of CS in the last 4 weeks, and it proved a strong sensitivity to change and recovery, with a cut-off score of 22 (22).

Regarding the pathogenesis, elevated impulsivity due to poor response inhibition was found to play an important role in experimental conditions, assessed by patients themselves or investigators, both in CS and in pathological gambling (PG) vs. healthy controls (2). Another trial (*N* = 103 patients with B/SD) supported a closer relationship between B/SD and BA than between B/SD and obsessive-compulsive symptoms, based on two delayed discounting tasks as markers of behavioral impulsivity (23). Mental disengagement could significantly predict vulnerability toward B/SD, according to a study based on an online questionnaire (*N* = 189 participants), which evaluated the main coping mechanisms used by these patients (24). Denial and substance use were also dysfunctional coping methods with predictive value for B/SD onset (24). A poorly developed, ambivalent, or contradictory self-image may create a vulnerable terrain for dysfunctional object attachment behaviors that may predict the B/SD onset (25). This disorder may be considered a chronic and repetitive failure in self-regulation, with cognitive, affective, and behavioral factors involved in different phases of the pathogenesis (26). Although these observations may have a significant impact on the therapy, there are many confounding factors that may limit their relevance for B/SD patients (e.g., research in laboratory settings vs. real-life conditions, lack of control for significant variables in the case of online questionnaires, or absence of culturally-defined variables that may contribute to the onset of B/SD in the explored studies). However, it is expected that the analysis of these outcomes (e.g., dysfunctional coping mechanisms, self-image distortions, and behavioral impulsivity) could be useful from the perspective of finding the most evidence-based therapies for patients with B/SD.

The neurobiological dimensions of B/SD have been hypothesized to involve serotoninergic, dopaminergic, and opioidergic neurotransmission, similar to other behavioral addictions, OCD, and substance use disorders (10). Based on these presumed mechanisms, the potential benefits of psychopharmacological agents (i.e., selective serotonin reuptake inhibitors-SSRIs, serotonin and norepinephrine reuptake inhibitors—SNRIs, opioid receptors antagonists—ORAs, or mood stabilizers) have been inferred. However, studies exploring the direct correlations between specific neurotransmitters or neuroanatomic pathways and the onset of B/SD are still lacking.

A genetic component of B/SD has been suggested, even if the available data support more of a vulnerability toward psychiatric disorders in first-degree relatives of B/SD patients than a specific tendency for CS (10). For example, CS/CB has been reported more frequently in descendants of individuals presenting with depression or substance use disorders (10).

In conclusion, the necessity of investigating the available treatments for B/SD is derived from multiple factors: the use of excessive buying as a way to cope with stress or isolation (27); negative functional consequences and risk of a chronic course (28); and the relatively high and increasing prevalence of problematic buying in the general population (1, 16). The need to assess the efficacy of psychotherapeutic, pharmacological and combined therapies in BAs or SUDs is also motivated by the high societal and personal costs associated with healthcare in B/SD, high risk of psychiatric or somatic comorbidity, and lower quality of life (29).

## Objectives

The main objective was to review the available data regarding the efficacy and tolerability of therapeutic interventions in the treatment of B/SD.

The second objective was to assess the validity of evidence-based recommendations, according to the GRADE system (30), for the therapeutic management of B/SD.

## Methodology

A systematic review was conducted in order to find the effects of therapeutic interventions dedicated to adult patients with B/SD. Primary and secondary reports, i.e., clinical reports, clinical and epidemiological studies, and reviews, were included in the analysis. The data extraction was guided by the PRISMA 2020 statements (31–33), and the corresponding steps are represented in Table 1. No automation tool was used in the process of data extraction.

**Table 1:** PRISMA 2020 checklist (31).

**Section and topic**	**Item #**	**Checklist item**	**The location where the item is reported**
**Title**			
Title	1	Identify the report as a systematic review.	Line 1
**Abstract**			
Abstract	2	See the PRISMA 2020 for the Abstracts checklist.	Lines 14–32
**Introduction**			
Rationale	3	Describe the rationale for the review in the context of existing knowledge.	Lines 133–141
Objectives	4	Provide an explicit statement of the objective(s) or question(s) the review addresses.	Lines 144–147
**Methods**			
Eligibility criteria	5	Specify the inclusion and exclusion criteria for the review and how studies were grouped for the syntheses.	Table 2, lines 173–176
Information sources	6	Specify all databases, registers, websites, organizations, reference lists, and other sources searched or consulted to identify studies. Specify the date when each source was last searched or consulted.	Lines 163–167
Search strategy	7	Present the full search strategies for all databases, registers, and websites, including any filters and limits used.	Lines 167–170
Selection process	8	Specify the methods used to decide whether a study met the inclusion criteria of the review, including how many reviewers screened each record and each report retrieved, whether they worked independently, and, if applicable, details of automation tools used in the process.	Lines 160–161
Data collection process	9	Specify the methods used to collect data from reports, including how many reviewers collected data from each report, whether they worked independently, any processes for obtaining or confirming data from study investigators, and, if applicable, details of automation tools used in the process.	Only one reviewer
Data items	10a	List and define all outcomes for which data were sought. Specify whether all results that were compatible with each outcome domain in each study were sought (e.g., for all measures, time points, analyses), and if not, the methods used to decide which results to collect.	Table 2
	10b	List and define all other variables for which data were sought (e.g., participant and intervention characteristics, funding sources). Describe any assumptions made about any missing or unclear information.	Table 2
Study risk of bias assessment	11	Specify the methods used to assess the risk of bias in the included studies, including details of the tool(s) used, how many reviewers assessed each study and whether they worked independently, and if applicable, details of automation tools used in the process.	Lines 182–187
Effect measures	12	Specify for each outcome the effect measure(s) (e.g., risk ratio, mean difference) used in the synthesis or presentation of results.	N/A
Synthesis methods	13a	Describe the processes used to decide which studies were eligible for each synthesis [e.g., tabulating the study intervention characteristics and comparing against the planned groups for each synthesis (item #5)].	N/A
	13b	Describe any methods required to prepare the data for presentation or synthesis, such as handling missing summary statistics or data conversions.	N/A
	13c	Describe any methods used to tabulate or visually display the results of individual studies and syntheses.	Table 3
	13d	Describe any methods used to synthesize results and provide a rationale for the choice(s). If meta-analysis was performed, describe the model(s), method(s) to identify the presence and extent of statistical heterogeneity, and software package(s) used.	N/A
	13e	Describe any methods used to explore possible causes of heterogeneity among study results (e.g., subgroup analysis, meta-regression).	N/A
	13f	Describe any sensitivity analyses conducted to assess the robustness of the synthesized results.	N/A
Reporting bias assessment	14	Describe any methods used to assess the risk of bias due to missing results in a synthesis (arising from reporting biases).	Table 4, lines 182–187
Certainty assessment	15	Describe any methods used to assess certainty (or confidence) in the body of evidence for an outcome.	Table 4, lines 182–187
**Results**			
Study selection	16a	Describe the results of the search and selection process, from the number of records identified in the search to the number of studies included in the review, ideally using a flow diagram.	Figure 2
	16b	Cite studies that might appear to meet the inclusion criteria but which were excluded, and explain why they were excluded.	Figure 2
Study characteristics	17	Cite each included study and present its characteristics.	Table 3
Risk of bias in studies	18	Present assessments of risk of bias for each included study.	Table 4
Results of individual studies	19	For all outcomes, present, for each study: (a) summary statistics for each group (where appropriate) and (b) an effect estimate and its precision (e.g., confidence/credible interval), ideally using structured tables or plots.	Table 3
Results of syntheses	20a	For each synthesis, briefly summarize the characteristics and risk of bias among contributing studies.	Lines 238–253, 309–315, and 339–345
	20b	Present results of all statistical syntheses conducted. If meta-analysis was done, present for each the summary estimate and its precision (e.g., confidence/credible interval) and measures of statistical heterogeneity. If comparing groups, describe the direction of the effect.	N/A
	20c	Present results of all investigations of possible causes of heterogeneity among study results.	N/A
	20d	Present results of all sensitivity analyses conducted to assess the robustness of the synthesized results.	N/A
Reporting biases	21	Present assessments of risk of bias due to missing results (arising from reporting biases) for each synthesis assessed.	Table 4
Certainty of evidence	22	Present assessments of certainty (or confidence) in the body of evidence for each outcome assessed.	Table 4
**Discussion**			
Discussion	23a	Provide a general interpretation of the results in the context of other evidence.	Lines 361–369
	23b	Discuss any limitations of the evidence included in the review.	Lines 373–382
	23c	Discuss any limitations of the review processes used.	Lines 373–382
	23d	Discuss the implications of the results for practice, policy, and future research.	Lines 383–389 and 395–398
**Other information**			
Registration and protocol	24a	Provide registration information for the review, including the register name and registration number, or state that the review was not registered.	N/A
	24b	Indicate where the review protocol can be accessed or state that a protocol was not prepared.	Lines 409–410
	24c	Describe and explain any amendments to the information provided at registration or in the protocol.	N/A
Support	25	Describe sources of financial or non-financial support for the review and the role of the funders or sponsors in the review.	Line 406
Competing interests	26	Declare any competing interests of review authors.	Lines 402–403
Availability of data, code and other materials	27	Report which of the following are publicly available and where they can be found: template data collection forms; data extracted from included studies; data used for all analyses; analytic code; any other materials used in the review.	N/A

### Design and research strategy

Five electronic databases (PubMed, PsychInfo, Cochrane, EMBASE, Clarivate/Web of Science) were included in the primary search. Also, the register of clinical trials run by the US National Library of Medicine (NLM) (www.clinicaltrials.gov) was explored for potential data regarding clinical trials dedicated to this topic. Also, websites and organizational websites dedicated to patients with B/SD were included in the sources exploration, and citation searching was also allowed.

The search paradigm used was “compulsive buying” OR “buying addiction” OR “shopping addiction” OR “shopping/buying dependence” AND “psychotherapy” OR “pharmacotherapy” OR “antidepressants” OR “mood stabilizers” OR “opioid antagonists” OR “medication”. All papers published between January 1990 and July 2022 were included in the primary search.

### Inclusion and exclusion criteria

Based on the SPIDER criteria (34), the retrieved sources were evaluated to see if they may be considered suitable for review (Table 2). The main sets of criteria referred to the characteristics of the sample, type of intervention, design of the research, methods of outcomes evaluation, and type of study (34).

**Table 2:** SPIDER algorithm for systematic reviews.

**Dimension**	**Inclusion criteria**	**Exclusion criteria**
Sample	Patients ≥18 years old were included.	Unspecified demographic parameters of the study population (clinical research).
	Patients were currently receiving at least one active intervention and/or placebo.	Lack of specifics about the therapeutic intervention administered.
	The presence of compulsive buying was defined as one of the main diagnoses in the explored population.	Insufficiently specified psychiatric diagnoses for clinical populations and/or compulsive buying was not specified as one of the main diagnoses in the recruited individuals.
Phenomenon of interest	The effects of short- or long-term psychotherapeutic or pharmacological intervention. Combined, psychotherapy and pharmacologic treatment was allowed.	Other interventions or not well-defined therapeutic strategies.
Design	Clinical trials, prospective or retrospective, randomized or not, controlled or not, single/double-blinded or unblinded.	Lack of well-defined design of the research.
	Case reports, systematic reviews, and meta-analyses.	Epidemiological studies.
		Research with different objectives (i.e., not including changes in compulsive buying or associated consequences) or contained poorly defined outcomes.
Evaluation	Efficacy and tolerability of therapeutic interventions explored population.	Lack of pre-defined measurements for the research's outcomes.
	Structured instruments administered by specialists and subjective reports.	Qualitative research, if they did not present clear references to the main objective of the review.
Research type	Qualitative and quantitative methods	Imprecise or loosely methodology of research.

### Study quality assessment

The quality of trials, case reports, and systematic reviews included in this analysis was assessed according to the Joanna Briggs Institute (JBI) critical appraisal checklists (57). These criteria were preferred because primary and secondary reports were allowed to enter the reviewing stage, and both qualitative and quantitative data were expected to result from the primary search. According to the JBI checklists, the sources may be included, excluded, or seeking further information is recommended (35, 36).

### Formulation of the therapeutic recommendations

The GRADE criteria for assessing the strength of recommendations based on the found evidence were applied to the final conclusions (31–33).

## Results

**Figure 2:**
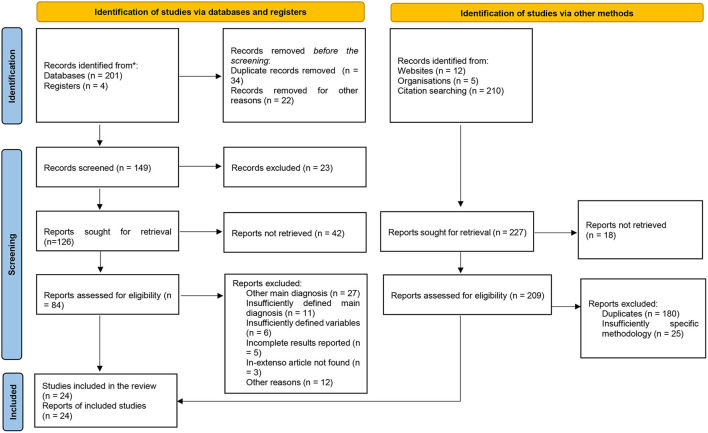
PRISMA 2020 flow diagram for systematic reviews (31).

Out of the initial 861 papers collected through the primary search, only 24 reached the final stage of selection (Figure 2). Eight case reports, 12 clinical studies, and four systematic reviews/meta-analyses were reviewed in detail, and the results are presented in Table 3. JBI Critical Appraisal Checklists for Case Reports, Randomized Studies, Quasi-experimental design studies, and Systematic Reviews were used in order to evaluate the quality of the research (35, 36).

**Table 3:** The main data reviewed for the evaluation of the efficacy and tolerability of therapeutic interventions in patients with B/SD.

**Authors**	**Design**	**Intervention**	**Outcomes**	**Results**	**Conclusions**
**Case reports**					
Kellett et al. (37)	Single case experimental study, cross-over design, 40-year female with B/SD, 350-day monitoring duration	Single CBT vs. PCE, CBT-13 out-patient sessions, then PCE- 6 out-patient sessions	Ideographic B/SD parameters were assessed *via* a diary, and nomothetic measures (CBS, CAS, BDI-II, IIP-32, BSI) were also used	The frequency and duration of CB episodes decreased during treatment. CBT and PCE were both effective vs. baseline. Shopping obsessions, excitement about shopping, and CS improved. Self-esteem also improved.	Both interventions were effective, but no significant differences were detected between them.
Marčinko and Kalovič (38)	Case report, a 32-year-old woman	Fluvoxamine and CBT, 52 weeks	The evolution of compulsive buying episodes	No compulsive buying episodes were reported at the follow-up (1-year).	No comorbid psychiatric disorders, but the assessment was of poor quality.
Marčinko et al. (39)	Two case reports, a 33 years old male, and a 41-year-old male, diagnosed with B/SD and BED	Fluvoxamine+ psychodynamic psychotherapy, 52 weeks	Pathological behaviors severity	Compulsive behavior decreased within 4 weeks and persisted at 52 weeks.	The BED comorbidity could influence the evolution of these patients.
Guzman et al. (40)	Case report, 37-year-old Caucasian woman with B/SD	Venlafaxine 225 mg/day plus topiramate 150 mg/day	Compulsive behavior, depressive symptoms	After 1-month compulsive behavior decreased in severity, and depression remitted.	The patient responded to a combination of SNRI and mood stabilizer, but there was no structured evaluation of the outcomes.
Ye et al. (41)	A 42-year-old woman diagnosed with type II BD, OCD, GAD, obesity, chronic trichotillomania, and B/SD. Six months of monitoring.	Topiramate was titrated to 100 mg/day and added to lamotrigine (50 mg QD) and aripiprazole (15 mg QD). The topiramate dose was further increased to 350 mg/day.	YBOCS-SV	Compulsive behavior has improved since day 4. YBOCS-SV score improved at week 6, declining from 31 (baseline) to 5. The compulsive behavior improvement was maintained after 6 months. Depressive/anxiety symptoms improved only when 300 mg/day of topiramate and 150 mg/day of lamotrigine were administered.	The compulsive behavior improved early, and it was not paralleled by depressive/ anxiety symptoms evolution.
Sepede et al. (42)	A 60-year-old woman, severe B/SD + MDD, SSRI-resistant, partially responsive to mirtazapine	Mirtazapine + bupropion	HAMA, HAMD, CGI, CBS, SCL-90-R, BIS-11	At week 28, the overall clinical status improved, with all the outcome measures significantly reduced with respect to baseline. No serious AE and only mild weight gain were recorded at the end-point.	The anti-craving and anti-withdrawal effects of bupropion may be explained by its anti-dopaminergic properties.
Braquehais et al. (43)	A 53-year-old male patient presented compulsive buying and collecting behaviors, alcohol use disorder, and obsessive-compulsive personality disorder.	Alcohol detoxification, followed by sertraline (100 mg/day) + topiramate (200 mg/day) + individual and group CBT-4 weeks hospitalization + follow-up for an unspecified period	Un-structured evaluation methods (no validated instruments were used); clinical observation was the only method of assessment	At the discharge, the therapy was considered successful because the patient reported a lower incidence of obsessive thoughts and lower debts acquired.	A pharmacological and psycho-therapeutical combination may lead to positive responses in patients with B/SD and multiple comorbidities.
Donahue et al. (44)	A 30-year-old patient	Motivational interview + imaginal desensitization	Self-reports	Increased self-control over buying behaviors, decrease in pathological impulses. At 6 months the improvement was stable.	A combination of behavioral, cognitive, psychoeducational, and motivational techniques may be useful in B/SD patients.
Di Nicola et al. (45)	Case report, a 42-year-old man diagnosed with B/SD, physical exercise addiction, and type I BD	Quetiapine titrated up to 600 mg/day was added to the maintenance treatment with divalproex 1,000 mg/day	CBS, EAI	Improvements in compulsive behaviors (CBS, EAI) were reported at week 12. The tolerability was good, and no significant AEs were reported.	This case signals the possible efficacy of adding quetiapine to the divalproex treatment, but the pathology of this patient is complex, with dual behavioral addiction and type I BD. Therefore, the efficacy of a certain drug over the B/SD, in this case, is difficult to delineate.
**Clinical trials**					
Filomensky and Tavares (46)	A clinical trial, *N* = 9 B/SD patients	20-week group CBT program	YBOCS-SV—cognitive, behavioral, and overall scores	All outcomes were significantly improved after CBT.	The results of the CBT therapy were favorable. However, the rate of comorbid mood disorders was high, and there was no control group.
Mitchell et al. (47)	A pilot trial, *N* = 28 patients with compulsive buying	Group CBT vs. waiting list	Number of compulsive buying episodes, time spent buying, YBOCS-SV, and CBS scores	CBT was associated with significant advantages in the ITT analysis vs. waiting list in all the outcomes, and the improvements persisted at 6 months.	CBT was efficient, but this was just an uncontrolled pilot study.
Müller et al. (48)	RCT, *N* = 56 patients with compulsive buying, 10 weeks for each intervention + 6-month follow-up	Group CBT vs. telephone guided self-help vs. waiting list	Primary outcome measure: CBS. Other outcome measures: YBOCS-SV, BDI	CBT was superior to the active comparator and to the placebo, but not significantly (primary outcome). Guided self-help had a superior efficacy when compared to the waiting list.	These results are preliminary, based on the small groups. No significant group-by-time interaction was found in the YBOCS-SV or BDI scores, but only in the CBS scores.
Mueller et al. (49)	*N* = 60 patients with B/SD, 12 weeks treatment + 6 months follow-up	CBT vs. waitlist control	The primary outcomes: YBOCS-SV, CBS, and German-CBS scores. Secondary outcomes: SCl-90-R, BIS-11, SI-R	The primary outcome-by-time-by-group effect was demonstrated between active and control groups. At 6-month the improvements were maintained in the primary outcomes. Other comorbidities were not affected by the treatment.	CBT could be effective vs. waitlist in the medium term.
Black et al. (50)	Open-label trial (*N* = 10 non-depressed patients with compulsive buying), 1-week placebo run-in + 9 weeks of active drug	Fluvoxamine up to 300 mg/day	CGI, YBOCS-SV	9 out of 10 patients became less interested in shopping, spent less time shopping, and they have lost less money in CS.	Fluvoxamine was efficient, but the trial was open-label and had a short duration.
Black et al. (51)	RCT, *N* = 23 patients with B/SD, 1-week single-blind placebo washout + 9 weeks active drug vs. placebo	Fluvoxamine vs. placebo	YBOCS-SV, CGI, HAMD, MOI	No significant differences between the active drug and placebo at the end of the trial. Both groups improved starting from week 2 and their favorable evolution persisted up to week 9.	Fluvoxamine did not distinguish itself from the placebo, and AEs were more frequent in the SSRI group (nausea, decreased motivation, sedation). This study evaluated treatments only in the short term.
Ninan et al. (52)	RCT, DB, 13-week, *N* = 23 B/SD patients who completed the study out of 42 screened	Fluvoxamine vs. placebo	YBOCS-SV, CGI, GAF, HAMD, patients' diaries	No significant differences were found between groups in any of the outcomes.	A high placebo rate was recorded in this study. However, fluvoxamine failed to distinguish itself from placebo.
Koran et al. (53)	A 1-week wash-out + a 7-week OL phase + a 9-week DB discontinuation phase, *N* = 26 adult patients with baseline YBOCS-SV ≥ 7	Escitalopram 10 mg/day OL, increased to 20 mg/day after four weeks	CGI-I, YBOCS-SV, MADRS	In the OL phase, YBOCS-SV decreased significantly (with almost 70%), and 19 patients were responders (by YBOCS-SV and CGI-I).	The DB phase did not confirm the OL escitalopram response. AEs were similar in the two groups during the DB phase.
Koran et al. (54)	A 7-week OL phase + a 9-week DB, placebo-controlled discontinuation phase, *N* = 24 patients with baseline YBOCS-SV ≥ 17	Citalopram 20 mg/day, gradually increased to 60 mg/day	YBOCS-SV, CGI-I	YBOCS-SV scores decreased significantly at week 7, with 15 patients being responders. Three discontinuations due to AEs (headache, rash, and insomnia). In the DB phase, five of the placebo-treated patients relapsed vs. none in the active drug group.	Citalopram was effective and well-tolerated for B/SD in the short term.
Koran et al. (55)	A 12-week OL trial, with follow-up visits for 12 months, *N* = 24 B/SD adult patients	Citalopram 20 mg/day initially, and increased every 2 weeks by 20 mg/day up to a maximum dose of 60 mg/day, depending on tolerability and responsivity.	YBOCS-SV, CGI-I	Citalopram led to marked, rapid, and sustained improvements on both scales. The response rate was 71%. Two patients were discontinued for AEs (sedation and agitation).	After 6 months, patients who continued citalopram therapy were less likely to relapse than those who discontinued the treatment. Therefore, both acute and long-term treatment with citalopram seems efficient.
De Mattos et al. (56)	RCT, DB, 12 weeks, *N* = 50 B/SD patients	Topiramate vs. placebo	Main outcome measure: YBOCS-SV. Secondary outcome scale: CB-FUS	No difference between groups in the drop-out rate or in the declining rate of B/SD symptoms (on YBOCS-SV). Topiramate significantly decreased the secondary outcome scores. Hoarding and impulsivity were reduced at a trend level in patients treated with topiramate.	The efficacy of topiramate vs. placebo was not supported by the primary outcome measure.
Grant et al. (57)	OL trial, *N* = 9 patients with B/SD, 10 weeks	Memantine (10-30 mg/day)	The primary outcome: YBOCS-SV	The YBOCS-SV score decreased significantly. Hours spent with CS and money spent decreased significantly. Impulsive buying and cognitive task performance improved, also.	The mean effective dose of memantine was 23.4 ± 8.1 mg/day. The overall tolerability was good, and its efficacy was confirmed at week 10. Still, this is an open-small-group group study.
**Systematic reviews and meta-analyses**					
Hague et al. (58)	A systematic review, *n* = 29 studies, only five were considered good-quality trials	Psychotherapy, pharmacotherapy	B/SD severity	Large effects were demonstrated for group psychotherapy and medication. Pharmacotherapy may improve outcomes in the long term in the B/SD population.	Group therapy is efficient, and pharmacotherapy may be an option due to its long-term favorable effects.
Leite et al. (59)	A systematic review, *n* = 23 articles	All types of interventions	B/SD severity	Only studies focused on CBT efficacy showed a significant response. Methodological flaws were found in the psychodynamically-oriented studies (no structured outcome assessment).	CBT may be the only efficient type of intervention. This conclusion was based mainly on case reports.
Soares et al. (60)	A systematic review of 21 studies	Pharmacological interventions	B/SD severity	Fluvoxamine was not efficient in PCTs (*n* = 3). Citalopram was efficient in OLTs. Escitalopram was effective in an OLT but not in the DB phase. Memantine was effective in a pilot OLT. Fluoxetine, bupropion, nortriptyline, clomipramine, topiramate, and naltrexone were effective in case reports.	Citalopram/ escitalopram may be effective, but the overall quality of the reviewed trials methodology was poor (mostly OLT).
Goslar et al. (61)	A meta-analysis focused on the treatment of Internet addiction, sex addiction, and B/SD (*n* = 91 studies, *N* = 3531 patients)	Psychotherapy, pharmacological treatment, and combined therapy	Structured and unstructured measurements of efficacy	Large-size pre-post reduction of the global severity of the B/SD symptoms by both psychological and pharmacological therapy. Psychological interventions were effective in decreasing compulsive behaviors especially if applied face-to-face and for longer periods of time. Combinations of CBT and medications led to advantages over monotherapies.	The therapy is effective in the short term, but the quality of most trials is poor.

AE, adverse events; BD, bipolar disorder; BDI, Beck Depression Inventory-II; BED, binge eating disorder; BIS-11, Barratt Impulsiveness Scale; B/SD, buying/shopping addiction; BSI, Brief Symptom Inventory; CAS, Compulsive Acquisition Scale; CB, compulsive buying; CBFUS, Compulsive Buying Follow-Up Scale; CBS, compulsive buying scale; CBT, cognitive-behavioral therapy; CGI-I, Clinical Global Impression-Improvement; CS, compulsive shopping; B/SD, compulsive shopping disorder; DB, double-blind; EAI, Exercise Addiction Scale; GAD, generalized anxiety disorder; GAF, Global Assessment of Functioning Scale; HAMA, Hamilton Anxiety Rating Scale; HAMD, Hamilton Depression Rating Scale; IIS-32, Inventory of Interpersonal Problems-32; ITT, intention-to-treat; MADRS, Montgomery-Asberg Depression Rating Scale; MDD, major depressive disorder; MOCI, Maudsley Obsessive-Compulsive Inventory; OCD, obsessive-compulsive disorder; OLT, open-label; PCE, person-centered experiential therapy; PCT, placebo clinical trials; RCT, randomized controlled trial; SCL-90-R, Symptom Checklist-90-Revised; SI-R, Saving Inventory-Revised; SNRI, serotonin and norepinephrine reuptake inhibitors; SSRI, selective serotonin reuptake inhibitors; YBOCS-SV, Yale-Brown Obsessive-Compulsive Scale- Shopping Version.

Table 4Assessment of the quality of evidence.

**Table:** 

**Case reports**									
**Source**	**Quality assessment criteria**								**Conclusion**
	**1**	**2**	**3**	**4**	**5**	**6**	**7**	**8**	
(18)	Yes	No	Yes	Yes	Yes	Yes	Yes	Yes	Include
(22)	Yes	No	Yes	Yes	Yes	Yes	No	Yes	Include
(23)	Yes	No	Yes	Yes	Yes	Yes	No	Yes	Include
(26)	Yes	No	Yes	Yes	Yes	Yes	Yes	Yes	Include
(32)	Yes	No	Yes	Yes	Yes	Yes	Yes	Yes	Include
(36)	Yes	No	Yes	Yes	Yes	Yes	Yes	Yes	Include
(38)	Yes	No	Yes	Yes	Yes	Yes	No	Yes	Include
(39)	Yes	No	Yes	Yes	Yes	Yes	No	Yes	Include
(43)	Yes	No	Yes	Yes	Yes	Yes	Yes	Yes	Include

**Table:** 

Quasi-experimental studies										
	Quality assessment criteria									Conclusion
	1	2	3	4	5	6	7	8	9	
(20)	Yes	N/A	N/A	No	No	Yes	N/A	Yes	Yes	Include
(24)	Yes	Yes	Yes	Yes	Yes	Yes	Yes	Yes	Yes	Include
(27)	Yes	N/A	N/A	No	No	Yes	N/A	Yes	Yes	Include
(31)	Yes	N/A	N/A	No	Yes	Yes	N/A	Yes	Yes	Include
(34)	Yes	Yes	Unclear	Yes	Yes	Yes	Yes	Yes	Yes	Include
(40)	Yes	N/A	N/A	No	Yes	Yes	Yes	Yes	Yes	Include
(41)	Yes	Yes	Unclear	Yes	Yes	Yes	Yes	Yes	Yes	Include

**Table:** 

Randomized clinical trials														
	Quality assessment criteria													Conclusion
	1	2	3	4	5	6	7	8	9	10	11	12	13	
(28)	Yes	Yes	Yes	Yes	Yes	Yes	Yes	Yes	Yes	Yes	Yes	Yes	Yes	Include
(29)	Yes	Yes	Yes	Yes	Yes	Yes	Yes	Yes	Yes	Yes	Yes	Yes	Yes	Include
(30)	Yes	Yes	Yes	Yes	Yes	Yes	Yes	Yes	Yes	Yes	Yes	Yes	Yes	Include
(33)	Yes	Yes	Yes	Yes	Yes	Yes	Yes	Yes	Yes	Yes	Yes	Yes	Yes	Include
(35)	Yes	Yes	Yes	Yes	Yes	Yes	Yes	Yes	Yes	Yes	Yes	Yes	Yes	Include

**Table:** 

Systematic reviews/meta-analyses												
	Quality assessment criteria											Conclusion
	1	2	3	4	5	6	7	8	9	10	11	
(19)	Yes	Yes	Yes	Yes	Yes	Yes	Yes	Yes	Yes	Yes	Yes	Include
(21)	Yes	Unclear	Yes	Yes	No	No	No	Unclear	Unclear	Yes	Yes	Include
(25)	Yes	Yes	Yes	Yes	No	No	No	Unclear	Unclear	Yes	Yes	Include
(42)	Yes	Yes	Yes	Yes	Yes	Yes	Yes	Yes	Yes	Yes	Yes	Include

### Case studies

A case study with a cross-over design that compared *cognitive-behavioral therapy* (CBT) and *person-centered experiential therapy* (PCE) in a 40-year-old B/SD patient found beneficial results for both interventions (37). These favorable effects were detected on ideographic (i.e., amount of money spent daily, time invested in B/SD, time spent on talking about buying, and B/SD-related cognitions and emotions) and nomothetic (i.e., scores on CBS, Compulsive Acquisition Scale, Beck Depression Inventory-II, Inventory of Interpersonal Problems-32, and Brief Symptom Inventory) outcomes related to B/SD severity, with no relapse over the follow-up period (99 days) (37). Another single-case report supports the therapeutic effect of *the fluvoxamine and CBT combination* in a female patient with B/SD during 52 weeks of unstructured monitoring, but no information about the duration of the psychotherapy or the antidepressant treatment was provided in this report (38). The same authors reported positive effects of *the fluvoxamine* (up to 150 and 200 mg daily, respectively) *and psychodynamically-oriented psychotherapy* (weekly sessions) in two female patients with B/SD and binge eating disorder (BED) after 3 weeks of combined treatment (39). No systematic methods were used to assess B/SD, but craving for buying and compulsive acts (e.g., shopping for large quantities of food, designer clothes or jewelry, acquiring antique objects, and compulsive eating) decreased based on patient self-report and clinician's observation (39).

*Topiramate* was explored as a potential treatment for B/SD in a 37-year-old female diagnosed with persistent B/SD and depression who was non-responsive to fluoxetine and venlafaxine. Topiramate was also selected based on previous reports of its efficacy in treating mood disorders and OCD (40). In this study, topiramate was added to venlafaxine up to 150 mg/day. The compulsive behavior (i.e., excessive shopping, mainly clothes) subsided in a month (but no objective measurement was provided by the authors), while depression was fully remitted (according to the Beck Depression Inventory scores) (40). Another case study explored the effect of topiramate (titrated up to 350 mg/day) as an augmenting agent in a 42-year-old female diagnosed with OCD, type II bipolar disorder, generalized anxiety disorder, obesity, trichotillomania, and B/SD (41). This patient also received lamotrigine and aripiprazole, which were added to topiramate, and the compulsive behaviors declined gradually for 6 months (according to the YBOCS-SV scores), with a slower pace of improvement for the anxiety and depressive manifestations (41).

The augmentation of *mirtazapine* (30 mg/day) with *bupropion* (300 mg/day) in a 60-year-old female patient diagnosed with a severe form of B/SD (i.e., high CBS score, spent family's money, ran into debt) and comorbid MDD led to clinical and psychometric improvements by week 8 (42). The compulsive behavior (assessed with CBS) remained significantly decreased at the end-point (week 28) (42). The assessment of the associated symptoms included the use of the Hamilton Anxiety Rating Scale (HAMA), Hamilton Depression Rating Scale (HAMD), Symptom Checklist-90-Revised (SCL-90-R), and Barratt Impulsiveness Scale (BIS-11) (42). All these scales recorded improvements (i.e., the severity of associated anxiety, depressive, and impulsive symptoms decreased). Also, the global overall clinical status, determined by the use of Clinical Global Impression (CGI), improved significantly at the final visit (42). The overall tolerability was good, with only a 4% increase in weight gain (42).

A combination of *sertraline, topiramate, and group and individual CBT* was associated with favorable results after discharge in a 53-year-old patient diagnosed with pathological buying and collecting behaviors, obsessive-compulsive personality disorder, and alcohol use disorder (43). The initial stage of the therapeutic management consisted of alcohol detoxification, followed by the combined intervention previously mentioned, that targeted both addictive and compulsive behaviors (43). No clinically validated scales were used to assess either hoarding or B/SD; the authors based their conclusions solely on clinical observation and patient reports (i.e., involvement in excessive shopping and hoarding, debt amount, and obsessions) (43).

*Motivational interviewing* and *imaginal desensitization* were successfully combined in a 30-year-old patient diagnosed with B/SD because they succeeded in increasing self-control over buying behaviors while decreasing the manifestation of pathological impulses (44). At 6 months, the improvement of the overall status was considered stable, assessed by clinicians' observations (44). Motivational interviewing helped the patient to develop intrinsic motivation for change, while the desensitization techniques were useful for confrontation and controlling impulsive behaviors (44).

*The combination of quetiapine and divalproex* was used as maintenance treatment in a 47-year-old male patient who presented with type I bipolar disorder comorbid with two behavioral addictions- B/SD and physical exercise addiction (45). After 12 weeks, the CS severity decreased under quetiapine (titrated up to 600 mg/day), according to the CBS scores, and the overall tolerability of this antipsychotic was good (45). The physical exercise addiction severity also decreased, according to the Exercise Addiction Inventory (EAI) scores (45).

Based on eight case reports, which evaluated the efficacy of pharmacological, psychotherapeutic, or combined interventions in nine patients presenting B/SD with psychiatric comorbidities (bipolar disorder *N* = 2, other BAs *N* = 2, personality disorders *N* = 1, substance use disorders *N* = 1, depressive disorders *N* = 2, OCD *N* = 1, anxiety disorders *N* = 1, impulse control disorders *N* = 1, eating disorders *N* = 1)/without such comorbidities (*N* = 1), the short-term prognosis of these individuals may be improved if treatment is initiated. The overall quality of data is low because there was no control group, not all reports have used structured and validated instruments for the monitoring of B/SD severity, concomitant medication was added in several cases, and there is a high degree of comorbidity, which may lead to a number of biases. Fluvoxamine combined with psychotherapy is supported by most data in this chapter, followed by various antidepressants (i.e., mirtazapine, venlafaxine, bupropion, and sertraline) either administered alone or in combinations, were associated with improved B/SD symptoms. Mood stabilizers (e.g., lamotrigine, topiramate, and divalproex) and atypical antipsychotics (i.e., aripiprazole and quetiapine) have been used especially in patients with B/SD and mood disorders, which make difficult to distinguish the impact of each disorder's impact over the clinical evolution. Very few reports on psychotherapy as the only intervention for these patients exist, therefore it is impossible to draw any conclusion on this topic for now.

### Clinical trials

A 20-week *group CBT program* was evaluated in a pilot study (*N* = 9 B/SD patients, mean age 41.8 years old) (46). Group therapy dedicated to the detection of specific shopping cognitive distortions and restructuring led to improvements in the cognitive, behavioral, and total scores of the YBOCS-SV after the therapy ended (46). Seven of the included patients currently presented with comorbid depression, and two of them had bipolar disorder, therefore, the results may be influenced by these comorbidities, which is impossible to establish without a control group (46).

The authors state, however, that “loss of control over shopping was not better explained by mood disorders since shopping bouts also occurred during periods of euthymia” (46).

A pilot study enrolled 28 compulsive buyers recruited through mass-media advertisements who subsequently received 12 sessions of group CBT over 10 weeks. Participants undergoing CBT had superior outcomes compared to the wait-list control in all the outcomes (both clinical variables and structured evaluation scores) at the end of treatment and 6-month follow-up (47). The main outcomes assessed were the number of compulsive buying episodes, time spent buying, YBOCS-SV, and CBS scores (47). Although this was just a pilot study, the results support the efficacy of group CBT on compulsive buying in outpatients with B/SD. Its main limitation is the absence of a comparison treatment, which prevents the causal correlation between this specific intervention (i.e., group CBT) and the outcome (i.e., improvement of the B/SD severity) (47).

Another pilot study included 56 patients with B/SD who were randomized on group CBT, telephone-guided self-help, or waiting list and monitored for the 10 weeks of the treatment, with a 6-month follow-up (48). Group CBT was superior to the self-help intervention and to the waiting list in reducing compulsive behaviors, and the favorable results were still present after 6 months (48). The CBS and YBOCS-SV scores declined significantly when compared to the baseline values for both active interventions, but the end-point differences between groups were not significant (48).

Another CBT trial focused on interruption and control of CS behaviors, training of healthy coping skills, and cognitive restructuring and enrolled 60 B/SD patients who were monitored for 12 weeks, with a 6-month follow-up (49). Significant improvement was detected in patients who received CBT vs. patients assigned to a waiting list at week 12, according to the primary outcome variables (YBOCS-SV, CBS, German-CBS) (49). Also, the improvement persisted at 6 months, but other psychopathology variables (i.e., compulsive hoarding, general psychopathology, impulsivity) were not significantly changed by the CBT vs. placebo (49).

A 9-week open-label trial with *fluvoxamine* (up to 300 mg/day) had a 1-week placebo run-in phase and included 10 non-depressed patients with CS (50). At the end of the trial, 9 patients presented improvements in their preoccupations, time spent, and money spent with these compulsive behaviors (50). Another 9-week trial, this time with a randomized, double-blind design following a 1-week single-blind placebo washout phase, did not find significant differences between fluvoxamine and placebo in any of the outcome measures, except for Maudsley Obsessive-Compulsive Inventory (MOCI) (51). In this second trial, fluvoxamine was associated with more adverse events than placebo, mainly nausea, insomnia/sedation, and decreased motivation (51). Yet another trial that evaluated fluvoxamine used a randomized, double-blind, placebo-controlled design and recruited 42 patients, out of which 23 completed the study after 13 weeks (52). No significant differences between groups were detected in any of the outcomes- YBOCS-SV, CGI, Global Assessment of Functioning (GAF), HAMD, and patients' self-reported compulsive behaviors (52).

In two trials conducted by the same team that used the same design (7-week open-label phase of active drug administration, followed by 9 weeks of double-blind, active drug vs. placebo), *citalopram* proved itself efficient in decreasing the YBOCS-SV scores and inducing higher response rate than placebo, but escitalopram was associated with favorable results only in the open-label phase (53, 54). However, these trials included small groups for each arm and relatively short durations of monitoring, therefore, their results should be interpreted with caution. In yet another trial, citalopram was administered open-label for 12 weeks in B/SD patients (*N* = 24), and it led to rapid, significant, and sustained improvements in both YBOCS-SV and CGI-I scores (55). Citalopram was associated with positive effects at 12 months of follow-up when compared to lack of treatment due to discontinuation (55).

A randomized, double-blind, controlled trial evaluated the efficacy of *topiramate* (up to 300 mg/day in the ninth week, if tolerated) vs. placebo for 12 weeks in 50 patients with B/SD (56). The superiority of topiramate vs. placebo was not confirmed by the main outcome measure (YBOCS-SV), and only clinical variables (hoarding, impulsivity) and Compulsive Buying Follow-Up Scale (CB-FUS) scores confirmed the efficacy of topiramate vs. placebo (56). However, the follow-up analysis suggested that topiramate may begin to distinguish itself from placebo after 10 weeks, which indicates the need for a longer duration of monitoring (56).

*Memantine* (23.4 ± 8.1 mg/day mean effective dose) improved the YBOCS-SV scores in a 10-week open-label trial that enrolled nine B/SD patients (57). The overall tolerability of memantine was good (57), but it should be mentioned that this was an open-label, small-group study, therefore, the efficacy results should be confirmed in larger trials.

According to the analysis of 12 clinical trials (*N* = 153 patients), mostly of moderate quality, group CBT benefits from consistent evidence of efficacy at 6 months, while the results supporting pharmacological interventions are scarce. Fluvoxamine led to negative results (*n* = 3 trials, *N* = 56 patients) on the main outcomes (i.e., CS severity and related variables, determined by either self-reported or clinician-rated scales) and possible low tolerability (reported in one trial), while citalopram and escitalopram (*N* = 74 participants) were associated with mixed results. Topiramate and memantine were evaluated only in one trial each (50 and nine patients, respectively), therefore, it is difficult to formulate clear conclusions about their efficacy in B/SD patients.

### Systematic reviews and meta-analyses

A systematic review of the *psychotherapy* (*n* = 17 studies) *and drug treatments*n=12 studies) concluded, based on mostly moderate and low-quality data (according to the criteria applied by the authors), that large effects were present for group psychotherapy and medication (58). Long-term treatment was correlated with better outcomes during pharmacotherapy but not when psychotherapy was administered (58).

Another review (*n* = 23 studies) concluded that *CBT* might be the only efficient method for the treatment of B/SD patients (59). However, this conclusion was based on case reports, and it must be mentioned that other psychotherapies did not use structured and validated instruments for the outcome measurements.

A review of 21 studies evaluating different pharmacological interventions found that placebo-controlled trials with *fluvoxamine* did not show effectiveness vs. placebo in B/SD patients, open-label trials with *citalopram* favored the active intervention vs. placebo, *escitalopram* was effective in an open-label trial, but not in the double-blind phase, and *memantine* was efficient only in a pilot open-label study (60). The authors of this review concluded that there is not enough evidence to support the recommendation of a specific agent for B/SD treatment (60).

In a meta-analysis dedicated to the pharmacological and psychological interventions for various behavioral addictions (Internet/sex/shopping dependence) that included 91 studies (*N* = 3531 participants), the results supported the efficacy of both types of therapy in the short-term (61). For B/SD, a large-sized pre-post decrease in the global severity of pathological behaviors was calculated: Hedge's *g* = 1.00 for psychotherapy and 1.52 for pharmacotherapy (61). *The combined psychotherapy and pharmacological approach* led to superior results to monotherapies, but the efficacy was demonstrated only in the short term (61).

The four systematic reviews/meta-analyses previously presented have formulated contradictory conclusions: while one found psychotherapy to be associated with the highest effect size (based on the results of 29 studies), another reported a superior effect size for pharmacotherapy (based on 91 studies) (58, 61). In the two reviews that evaluated only psychotherapeutic interventions and only pharmacotherapy respectively (based on 23 and 21 studies), CBT was considered the only efficient method (59), while no pharmacological agent could be recommended yet as monotherapy for B/SD patients (60).

## Conclusions

B/SD is a very complex pathology, which integrates elements from BAs, OCD spectrum, and impulse control disorders, which raises the question of the heterogeneity of the populations included in the reviewed reports. It could be conceived that some of them are more close to a BA, some of them are more impulsive, while still others associate elements from the OCD spectrum. Various combinations of these dimensions in the same patient are also theoretically possible, and the high rate of comorbidity reported in this population may support this perspective.

Based on reviewing 24 distinct sources, representing case reports (*n* = 8), clinical trials (*n* = 12), and systematic reviews/meta-analyses (*n* = 4), it may be concluded that psychotherapy, and especially group CBT may be recommended for B/SD patients (supported by the results of two reviews, one case study, and four clinical trials) (Table 5). Combined, pharmacotherapy and psychotherapy, may be recommended, but the data in favor of this strategy is less significant (supported by three case reports, and one meta-analysis). No specific recommendations for pharmacological agents could be made, although positive results with serotonergic antidepressants and topiramate exist. In patients with associated mood disorders, mood stabilizers (topiramate, lamotrigine, divalproex) and atypical antipsychotics (aripiprazole, quetiapine) have been correlated with positive results in case studies.

**Table 5:** Summary of GRADE recommendations for B/SD treatment.

**Therapeutic intervention**	**GRADE recommendations**	**Supporting reports**	**Observations**
Cognitive-behavioral therapy	**B+**	(37, 46–49, 59, 61)	Most of the explored studies are short-term. Supporting data are derived from case reports and poor- to moderate-quality trials.
Pharmacotherapy plus psychotherapy	**B** for BS/D **B+** in patients with psychiatric comorbidities	(38, 39, 43, 61)	The positive effects of this strategy were demonstrated in the short term.
Serotonergic antidepressants	**C** for B/SD **C+** for patients with B/SD and mood disorders	(50, 54, 55, 60)	Escitalopram/citalopram may be efficient, but the reviewed trials were of low quality.
Moodstabilizers	**D** for B/SD **C** for patients with B/SD and mood disorders	(40, 41, 56)	Topiramate seems promising, but larger trials are needed. The association of venlafaxine to topiramate has been also associated with positive results in a case report.
Memantine	**C–**	(57)	Only one trial support this intervention.
Atypical antipsychotics	**C–** for patients with dual diagnosis, B/SD and mood disorder	(45)	Quetiapine may be efficient as an add-on in this specific population. However, data to support its efficacy is very limited.
Motivational interview plus imaginal desensitization	**D+**	(44)	Low level of support for this combination.

The GRADE recommendations formulated were A (high), B (moderate), C (low), or D (very low) (42, 55), according to the level of confidence that therapeutic interventions will improve the outcome of patients with B/SD.

The strengths of the current review rely on the inclusion of both primary and secondary reports detected through a systematic literature search and on the formulation of evidence-based recommendations with potential clinical utility. The reports were assessed for methodological quality using validated checklists (JBI), and the recommendations were made in accordance with GRADE criteria.

As limitations of the current review, it must be mentioned that conclusions integrated data derived from studies with multiple comorbidities, but since not all the researchers have made a thorough screening for psychiatric comorbidity at baseline, it is possible that other pathologies might be escaped, and yet influenced the outcomes. Due to the high rate of dual diagnosis in B/SD, screening for other psychiatric disorders is needed initially and periodically in this population. The high rate of SUDs and behavioral addictions with other psychiatric disorders has been reported in many sources (1, 15, 18, 19, 36, 62). It is difficult to interpret data resulting from the treatment of patients with multiple comorbidities, especially in case reports. The quality of the reviewed data is heterogenous, with case reports not using structured methods of monitoring, and a short duration of observation. Another limitation derives from the fact that data extraction and quality assessment were conducted by only one researcher.

Regarding the specifics of the recommendations for patients with B/SD, fewer sessions of group therapy and more severe pre-treatment hoarding features were significant predictors for nonresponse to the CBT (63). The risk of poor adherence to the individual CBT program was 28% in a study that enrolled 97 B/SD patients (63). Also, a significant discontinuation rate of 46.4% was reported, and the predictors of poor therapy adherence were male gender, more severe depression and obsessive-compulsive symptoms, lower anxiety level, high persistence, high harm avoidance, and low self-transcendence (63).

The prognosis of B/SD management is dependent on the adequate treatment of comorbid psychiatric conditions, psychological vulnerability factors, and sufficient time for monitoring. Therefore, an initial comprehensive evaluation of the patients presenting CS/CB is granted.

It is expected that further research will evaluate larger populations and more homogeneous participants. Also, a longer duration of clinical trials is needed, in order to confirm the efficacy of therapeutic interventions in patients with a known high rate of relapse.

## Data availability statement

The original contributions presented in the study are included in the article/supplementary material, further inquiries can be directed to the corresponding author.

## Author contributions

OV contributed to the article and approved the submitted version.

## Conflict of interest

The author declares that the research was conducted in the absence of any commercial or financial relationships that could be construed as a potential conflict of interest.

## Publisher's note

All claims expressed in this article are solely those of the authors and do not necessarily represent those of their affiliated organizations, or those of the publisher, the editors and the reviewers. Any product that may be evaluated in this article, or claim that may be made by its manufacturer, is not guaranteed or endorsed by the publisher.
